# Novel insights into axon diameter and myelin content in late childhood and adolescence

**DOI:** 10.1093/cercor/bhac515

**Published:** 2023-01-04

**Authors:** Sila Genc, Erika P Raven, Mark Drakesmith, Sarah-Jayne Blakemore, Derek K Jones

**Affiliations:** Cardiff University Brain Research Imaging Centre (CUBRIC), School of Psychology, Cardiff University, Maindy Rd, Cardiff CF24 4HQ, United Kingdom; Cardiff University Brain Research Imaging Centre (CUBRIC), School of Psychology, Cardiff University, Maindy Rd, Cardiff CF24 4HQ, United Kingdom; Department of Radiology, New York University School of Medicine, 550 1st Ave., New York, NY 10016, United States; Cardiff University Brain Research Imaging Centre (CUBRIC), School of Psychology, Cardiff University, Maindy Rd, Cardiff CF24 4HQ, United Kingdom; Department of Psychology, University of Cambridge, Downing Pl, Cambridge CB2 3EB, United Kingdom; Cardiff University Brain Research Imaging Centre (CUBRIC), School of Psychology, Cardiff University, Maindy Rd, Cardiff CF24 4HQ, United Kingdom

**Keywords:** development, microstructure, MRI, puberty, diffusion

## Abstract

White matter microstructural development in late childhood and adolescence is driven predominantly by increasing axon density and myelin thickness. *Ex vivo* studies suggest that the increase in axon diameter drives developmental increases in axon density observed with pubertal onset. In this cross-sectional study, 50 typically developing participants aged 8–18 years were scanned using an ultra-strong gradient magnetic resonance imaging scanner. Microstructural properties, including apparent axon diameter $({d}_a)$, myelin content, and g-ratio, were estimated in regions of the corpus callosum. We observed age-related differences in ${d}_a$, myelin content, and g-ratio. In early puberty, males had larger ${d}_a$ in the splenium and lower myelin content in the genu and body of the corpus callosum, compared with females. Overall, this work provides novel insights into developmental, pubertal, and cognitive correlates of individual differences in apparent axon diameter and myelin content in the developing human brain.

## Introduction

The human brain undergoes significant remodeling across childhood and adolescence (Mills et al. 2016). Studies using magnetic resonance imaging (MRI) have demonstrated that white matter undergoes rapid maturation in early childhood (Hagmann et al. 2010; Tamnes et al. 2018; Reynolds et al. 2019), followed by a more steady development of tissue properties along the posterior to anterior maturation gradient into adolescence (Lebel and Beaulieu 2011; Bartzokis et al. 2012; Palmer et al. 2022).

Differences in pubertal development may critically modulate age- and sex-dependent maturation of brain structure (Blakemore et al. 2010). Previous work suggests that the activation of the hypothalamic–pituitary–adrenal (HPA) axis in childhood triggers a cascade of neuronal development (Sisk and Foster 2004; Byrne et al. 2017), mediated by the rise in adrenal hormones (Maninger et al. 2009). This earliest stage of pubertal development, termed adrenarche, is hallmarked by a rapid increase in adrenal hormones at 6–8 years of age in females and approximately 1–2 years later in males (Grumbach and Styne 1998; Dorn et al. 2006). The next stage in the pubertal time course is the period of gonadarche, which begins at around 9–10 years of age in girls and approximately 1 year later in boys. Sex steroid hormones, such as estradiol and testosterone, are modulated by the activation and feedback mechanism of the hypothalamic-pituitary-gonadal (HPG) axis. Sex steroid hormones can cross the blood–brain barrier and act as trophic factors impacting the development of axons and supporting cells in the brain (Sisk and Foster 2004). These sex differences in pubertal development (both with respect to adrenarche and gonadarche) may influence brain morphometric (Peper et al. 2009; Perrin et al. 2009; Chavarria et al. 2014) and microstructural properties (Herting et al. 2012; Herting et al. 2017; Genc, Smith, et al. 2018b).

*Ex vivo* studies in the macaque brain reveal that axon count (i.e. total number of axons) in the corpus callosum stabilizes in the early post-natal period (LaMantia and Rakic 1990), suggesting that white matter maturation in childhood and adolescence is driven by coupled radial growth of the axon and myelin sheath (Yakovlev and Lecours 1967; Paus 2010; Berman et al. 2017). Critically, rodent models have revealed sex- and puberty-related mediation of axon diameter dynamics (Kim and Juraska 1997; Pesaresi et al. 2015), g-ratio (Pesaresi et al. 2015; Stikov et al. 2015), and myelin content (Nuñez et al. 2000) in the splenium of the corpus callosum. Based on this cumulative evidence, our primary hypothesis was that age-related and pubertal increases in callosal axon density (Genc, Smith, et al. 2018b) would be driven by radial axonal growth. As age and pubertal stage are highly correlated in early adolescence, brain–hormone interactions may be key in identifying critical periods of growth in axonal diameter and myelin.

The axon diameter (the internal diameter of the axon, *d_a_*) is positively associated with conduction velocity (Gasser and Grundfest 1939), and the myelin thickness further modulates conduction velocity, but there is not a monotonic relationship between myelin thickness and conduction velocity. Rather, the ratio of the inner diameter (*d*) to the outer diameter of the myelinated axon (*D*), named the g-ratio (g = *d*/*D*), influences the conduction velocity, with Rushton (1951) being the first to derive a theoretical optimal value of 0.6. While a number of biophysical and electrophysiological properties of white matter influence neuronal conduction, it is axon diameter that explains the greatest proportion of variance in conduction velocity (Drakesmith et al. 2019), rendering this parameter of greater interest for investigating links between brain microstructure and specific aspects of cognitive function where conduction velocity is likely to be critical, for example, for processing speed.

Concurrent to the maturation of white matter microstructure is the development of executive functions (Luna et al. 2015; Larsen and Luna 2018; Fuhrmann et al. 2020; Goddings et al. 2020), which can be considered functional sequelae of microstructural changes. The relationship between microstructural development and executive functions has been explored in the literature, largely using the technique of diffusion tensor MRI, which uses a simplistic 3-dimensional representation of the diffusion-weighted MRI signal to infer on measures such as the fractional anisotropy (FA; see Box 1 for further interpretation). For example, working memory capacity and processing speed have both been associated with developmental differences in FA, suggesting that white matter microstructural changes underpin the improvement of these functions (Fryer et al. 2008; Ferrer et al. 2013; Krogsrud et al. 2018). Here, in preliminary data from a small subset of participants, we focus on working memory as a facet of executive function. The underlying microstructural attributes that contribute to these differences in FA (e.g. myelination, axon density, axon diameter, axonal orientation dispersion [OD]) can be further explored using alternative methods (Jones et al. 2013).

**Box 1:** Description of microstructural measures.

Measure	Abbreviation	Interpretation
Fractional anisotropy	FA	A measure that is scaled between zero and one that quantifies the extent to which diffusion is anisotropic in the image voxel. A value of zero suggests that the rate of diffusion is the same in all directions (isotropic), whereas as a value of 1 would indicate that diffusion is constrained to be non-zero along a single orientation. This measure is derived from diffusion tensor MRI
Orientation dispersion	OD	A measure of the extent to which neurites (axons) are aligned along a single axis. If all axons in the voxel are co-axial, then the OD is zero. If they are uniformly oriented in 3-dimensional space, then the OD is 1. This measure is derived from multi-shell diffusion MRI acquisitions and by applying models such as “NODDI” (Zhang et al. 2012)
Restricted diffusion signal fraction	FR	Derived from multi-compartment models of tissue microstructure, this is the fraction of the signal that comes from water inside a restricted space. In the case of white matter, this is assumed to be intra-axonal water. Thus, the “restricted diffusion signal fraction” reflects the fraction of signal from water inside the axon. This measure is therefore often interpreted as the “axon density”
Axon diameter	*d_a_*	This is the estimate of the inner diameter of axons as derived from diffusion MRI-based modeling
Magnetization transfer saturation	MTSat	A semi-quantitative index of myelin content based on the magnetization transfer ratio (MTR), which is additionally corrected to remove effects of longitudinal relaxation time, T_1_, that are known to oppose and thus reduce MT contrast
g-ratio	*g*	The ratio of the inner to the outer diameter of the myelin sheath, computed from a mixture of diffusion-MRI and myelin sensitive acquisitions
Conduction velocity	cv	This is the estimate of axonal conduction velocity in white matter estimated from MR-derived estimates of g-ratio, *g*, and axon diameter, *d_a_*

There has been considerable progress over recent years in microstructural acquisition and modeling approaches to quantify these separate aspects of tissue microstructure. For example, early works reported on *in vivo*axon diameter estimates (Assaf et al. 2008; Barazany et al. 2009; Alexander et al. 2010). *In vivo* estimation of axon diameter, however, can be challenging, as the lower bound on axon diameter estimates with most MRI systems is approximately 4 μm (Nilsson et al. 2017), whereas a large proportion of the adult corpus callosum is made up of axons 1–2 μm in diameter (Aboitiz et al. 1992). This lower bound on axon diameter estimates is driven principally by the maximum gradient strength available on the MRI scanner, with most state-of-the-art systems providing 80 mT/m. Moreover, the recent availability of ultra-strong gradients (300 mT/m) (McNab et al. 2013; Jones et al. 2018) has facilitated the improvement of axon diameter estimates *in vivo* (Drakesmith et al. 2019; Huang et al. 2019; Veraart et al. 2020).

Diffusion MRI (dMRI), however, and particularly FA, is only weakly sensitive to differences in myelin (Beaulieu 2002). While no MRI-based measurement can be considered a “pure” measurement of myelin (see, e.g. Mancini et al. 2021), measures based on magnetization transfer contrast (Wolff and Balaban 1989) show the highest correlation with myelin content (Mancini et al. 2021). The “magnetization transfer ratio” (MTR) is the most widely used measure of magnetization transfer, correlating positively with myelin content, but it is recognized that differences in other factors (including the longitudinal relaxation time, *T*_1_) can add unwanted variance to the measurement. Magnetization transfer saturation (MTsat) is a semi-quantitative measure, with only modest increased demand on data acquisition compared to MTR, but which removes the dependence on *T*_1_. Finally, recent reports show how to compute the aforementioned g-ratio from MT-based and diffusion-based MRI measurements (Campbell et al. 2018). A summary of these MRI-derived measures is presented in Box 1.

In this study, we apply ultra-strong gradient MRI to study developmental patterns of axon and myelin microstructure in a cross-sectional sample of typically developing participants aged 8–18 years. Based on converging *in vivo* and *ex vivo* evidence, our primary aim was to show that developmental profiles of apparent axon diameter, myelin content, and g-ratio provide novel and specific insights into age-, sex-, and puberty-mediated differences in microstructure. Our secondary aim was to explore the relationship between microstructural predictors of axonal conduction velocity and working memory performance in a small subsample.

## Methods

### Participants

This study reports on a subsample of typically developing participants aged 8–18 years (*N* = 50; mean age = 13.5, SD = 2.9 years; 30 female) recruited as part of the Cardiff University Brain Research Imaging Centre (CUBRIC) Kids study (Genc et al. 2020). The subsample comprised participants that underwent a dedicated axon diameter mapping protocol (detailed in Image acquisition section). The study was approved by the School of Psychology ethics committee at Cardiff University. Participants and their parents/guardians were recruited via community and public outreach events. Written informed consent was provided by the primary caregiver, and adolescents aged 16–18 years additionally provided written consent. Participants were excluded from the study if they had non-removable metal implants, and/or if they reported history of a major head injury or epilepsy. Participants were excluded from the current study if the parent/guardian reported a diagnosis of a neurodevelopmental or psychiatric condition.

### Image acquisition

Participants were scanned on a 3T Siemens Connectom MRI system with ultra-strong (300 mT/m) gradients.

Structural T_1_ -weighted imaging data were acquired using a 3D Magnetization-Prepared Rapid Gradient Echo (MPRAGE) protocol with 1 × 1 × 1 mm voxel size; echo time (TE)/repetition time (TR) = 2/2,300 ms; matrix = 256 × 256, 192 slices.

Multi-shell dMRI data were acquired using a (1) standard multi-shell acquisition protocol and (2) variable diffusion time protocol for whole-brain axon diameter mapping, or (3) a modified version of (2) which had a smaller field of view covering the corpus callosum to shorten the overall acquisition time, as follows:

(1) Fixed diffusion time: TE/TR = 59/3,000 ms; voxel size = 2 × 2 × 2 mm; matrix = 110 × 110, 66 slices, *b*-values = 0 s/mm^2^ (14 volumes, interleaved), 500 s/mm^2^ (30 directions), 1,200 s/mm^2^ (30 directions), 2400 s/mm^2^ (60 directions), 4,000 s/mm^2^ (60 directions), and 6,000 s/mm^2^ (60 directions), δ = 7 ms, Δ = 23 ms.
(2) Variable diffusion time: TE/TR = 80/3,900 ms; voxel-size 2 × 2 × 2 mm; matrix = 110 × 110, 66 slices, *b*-values = 0 s/mm^2^ (4 volumes), 2,000 s/mm^2^ (30 directions); 4,000 s/mm^2^ (60 directions); δ = 7 ms. Acquisition was repeated over 4 separate diffusion times: Δ = [18,30,42,55] ms.
(3) Variable diffusion time with cropped field of view (FoV): TE/TR: 80/2,200 ms; voxel-size 2 × 2 × 2 mm; matrix = 110 × 110, 30 slices, *b*-values = 0 s/mm^2^ (4 volumes), 2,000 s/mm^2^ (30 directions); 4,000 s/mm^2^ (60 directions); δ = 7 ms. Acquisition was repeated over 4 separate diffusion times: Δ = [18,30,42,55] ms.

All dMRI data were acquired with the phase encoding in an anterior–posterior (AP) direction, with 2 additional PA-encoded volumes.

 Multi-parametric mapping data were acquired with the following parameters: Three multi-echo 3D FLASH (TE = 2.46–19.68, ES = 2.46, voxel-size = 1.5 × 1.5 × 1.5 mm) scans were acquired with either T_1_-, proton density (PD-), or magnetization transfer-weighting (MTw) by varying the TR and flip angle, α, or 23 ms/28°, 23 ms/5°, 42 ms/7°, respectively. For MTw, an off-resonance Gaussian RF pulse was applied prior to excitation (Helms et al. 2008; Weiskopf et al. 2011).

Supplementary Table S1 summarizes the imaging protocol duration and total number of participants completing each sequence.

### Preprocessing and model fitting

Diffusion MRI data were preprocessed using a combination of FSL (Smith et al. 2004), MRtrix3 (Tournier et al. 2019), and in-house software, as follows: correction for signal drift (Vos et al. 2017); motion, eddy, and susceptibility-induced distortions (Andersson and Sotiropoulos 2016); gradient non-linearities (Rudrapatna et al. 2020); and Gibbs ringing artifact (Kellner et al. 2016). Each individual’s diffusion data were then registered to their skull-stripped structural T_1_-weighted image. Root mean squared (RMS) displacement estimated from *eddy* (Andersson and Sotiropoulos 2016) was used as a summary measure of global head motion.

FA was computed by fitting the diffusion tensor (Basser and Pierpaoli 1996). The neurite orientation dispersion and density imaging (NODDI) model (Zhang et al. 2012) was used to obtain an estimate of intracellular volume (We note that these volume fractions are more correctly referred to as signal fractions. However, we have chosen to retain the original nomenclature for ease of comparison with previous studies.) fraction (${v}_{ic}$), OD, and isotropic volume^*^ fraction (${v}_{iso}$) using all *b*-values from protocol (1).

 Apparent axon diameter (${d}_a$), in μm, was computed using AxCaliber models (Assaf et al. 2008) fitted to the variable diffusion time data using microstructure diffusion toolbox (MDT) (Harms et al. 2017). A cascaded modeling approach was used to initialize the AxCaliber model fit. First, data were fit to a ball and stick model (Behrens et al. 2003) to provide axon orientation estimates which were then fixed. Next, the restricted diffusion signal fraction (FR) and diffusivity were derived from the composite hindered and restricted model of diffusion (CHARMED) model (Assaf and Basser 2005), which served as initial values for the AxCaliber model. In this model, the ${d}_a$ distribution was modeled by a continuous Poisson distribution with a time-dependent zeppelin modeling the extracellular space (De Santis et al. 2016), using both the full and truncated gamma distributions (Drakesmith et al. 2018).

 Magnetization transfer data were corrected for bias receive field artifacts and Gibbs ringing. MT data corrected for T_1_ effects (MTSat) were generated using the QUIT toolbox (Wood 2018). For each participant, MT-weighted volumes were aligned to their T_1_-weighted image co-registered to diffusion space for motion correction.

The g-ratio, the ratio of the inner to the outer diameter of the myelin sheath (Rushton 1951), was estimated by using previously derived equations and calibration factors to generate axonal and myelin volume fractions (AVF and MVF), respectively (Stikov et al. 2015; Ellerbrock and Mohammadi 2018; Drakesmith et al. 2019), where:


$$ g=\sqrt{\frac{1}{1+\frac{\mathrm{MVF}}{\mathrm{AVF}}}} $$

$$ \mathrm{MVF}=0.11\times \mathrm{MTSat} $$

with the AVF estimated using the restricted diffusion signal fraction from the CHARMED model:


$$ \mathrm{AVF}=\left(1-\mathrm{MVF}\right)\times \mathrm{FR} $$

or from the NODDI model,


$$ \mathrm{AVF}=\left(1-\mathrm{MVF}\right)\left(1-{v}_{iso}\right)\times{v}_{ic} $$

Predicted axonal conduction velocity $(cv)$, in m/s, was derived using voxel-wise estimates of apparent axon diameter and g-ratio, as previously described by Drakesmith et al. (2019):


$$ cv=p{d}_a\sqrt{-\log (g)} $$

Here *p*, the proportionality constant, was set to 16.99:


$$ cv=16.99\times{d}_a\times \sqrt{-\log (g)} $$

**Fig. 1:**
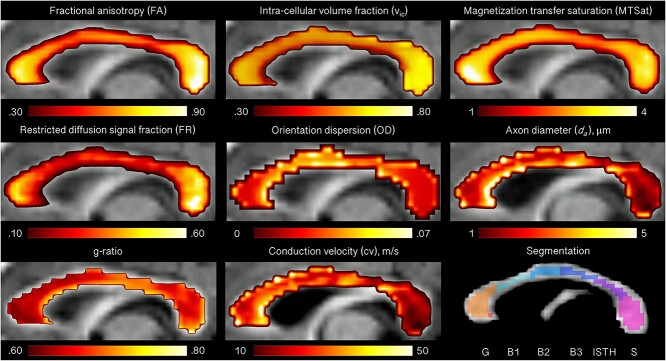
Microstructural maps for one representative participant (11 year old male). The corpus callosum was segmented into 6 regions: the genu (G), body (3 segments; B1, B2, B3), isthmus (ISTH) and splenium (S).

The mid-sagittal slice of the corpus callosum was manually segmented for each participant. A 6-region parcellation (G: genu; B1: anterior body; B2: mid body; B3: posterior body; ISTH: isthmus; S: splenium) was performed using a template (Witelson 1989), transformed to subject-specific maps, and manually edited to exclude voxels with partial volume artifacts. Representative microstructural maps are presented in Fig. 1.

Following quality control of MRI data, a total of 48 participants with dMRI data and 34 participants with MT data were used for subsequent statistical analyses.

### Measures

Participant characteristics are summarized in Table 1. Height and weight were determined using the average of 2 consecutive measurements and used to calculate a body mass index (BMI) in kg/m^2^. The Strengths and Difficulties Questionnaire (SDQ; Goodman (1997)) was administered as a survey to the primary caregiver of all participants, as well as directly to participants aged 11 and above. A total difficulties score was computed using all scales except the prosocial scale as a summary measure of emotional/behavioral difficulties.

**Table 1:** Participant characteristics and their relationship (*R*^2^) with age (*N* = 50).

**Measure**		**Mean**	**SD**	***R*** ^**2**^	***P-*value**
Age, years		13.47	2.95		
Pubertal stage					
	PDSS	3.26	1.50	0.75	<0.001
	PDSA	3.08	1.51	0.72	<0.001
	PDSG	2.98	1.56	0.76	<0.001
Body mass index, kg/m^2^		19.87	3.19	0.27	<0.001
SDQ, total score		5.92	3.62	0.02	0.98
Working memory^a^					
	Hit reaction time, ms	1102	236	0.59	0.004
	d prime	1.99	0.81	0.10	0.18
Motion, RMS displacement					
	dMRI, protocol 1	1.43	0.87	−0.02	0.58
	dMRI, protocol 2	1.73	1.25	0.06	0.07
	dMRI, protocol 3	1.46	0.49	−0.01	0.37

Note: Statistics (adjusted *R*^2^, *P*-value) summarize age associations from a linear model. The range of all PDS scores was 1–5. dMRI, diffusion magnetic resonance imaging; PDS, Pubertal Development Scale; PDSA, adrenal PDS; PDSG, gonadal PDS; PDSS, total PDS; RMS, root mean square; SD, standard deviation; SDQ, Strengths and Difficulties Questionnaire. ^a^Assessed in a subset of 11 participants.

### Pubertal maturation

The Pubertal Development Scale (PDS; Petersen et al. (1988)) was administered as a survey to the primary caregiver of all participants, as well as directly to participants aged 11 years and above. Physical development was rated on a 4-point scale. This included questions assessing the presence of characteristics phenotypical of pubertal onset such as deepening of voice and presence of facial hair in males, and breast development and menarche for females, as well as skin changes and pubic hair growth in both males and females. Three PDS scores were generated: an adrenal score (PDSA), which summarizes features of adrenarche; a gonadal score (PDSG), which summarizes features of gonadarche; and a total score (PDSS), which combines features of adrenarche and gonadarche (Shirtcliff et al. 2009). Gender was determined by parent and self-report. To aid with interpretations around the neurobiological actions of sex hormones, we herein refer to gender as sex. Pubertal stage was further divided into prepubertal (no evidence of maturation of secondary sexual characteristics) and pubertal (evidence of pubertal maturation).

### Cognitive assessment

Directly following the MRI session, 11 participants aged 8–18 years (Mean age = 13.08 years, 4 female) who underwent dMRI protocol (3) were administered an Emotional N-back task of working memory on an iPad. On each trial, participants were told to respond indicating whether or not each image matched the image shown 2 images prior (2-back). Four blocks of 2 conditions were run, one with emotional stimuli (happy, fearful, and sad faces) and one neutral block with shapes. The 2 conditions (emotional 2-back and neutral shapes 2-back) were run in randomized order for all participants. For the purposes of the current study, only data from the neutral shapes condition were analyzed.

The primary measure of interest was hit reaction time (in ms), an indicator of cognitive processing speed, captured when the participant scored a hit (i.e. correctly identified a target) (Forns et al. 2014). The secondary measure of interest was *d* prime (${d}^{\prime}$), an indicator of working memory capacity, estimated using the relative proportion of hits minus false alarms (Haatveit et al. 2010). Given the small sample size of participants with cognitive data, analyses including these data were considered exploratory.

### Statistical analyses

All statistical analyses and data visualizations were performed in R (v3.4.3). To investigate region and measure-specific relationships with respect to developmental characteristics, we performed linear mixed effects modeling using *lme4* and *lmerTest*. The primary advantage of using mixed effects modeling in neuroimaging studies is that it allows for hierarchical structures observed in data with repeated measures and appropriately handles missing data (across contrasts and across varying voxel populations). Voxel-wise data in the upper (99%) and lower (1%) quantiles were trimmed to exclude potential outliers. To confirm that our findings were not influenced by relative differences in head motion, the main analyses were repeated including RMS displacement as a predictor.

We sought to determine:

- Regional patterns of microstructural measures (FA, OD, ${v}_{ic}$, FR, ${d}_a$, MTSat, g-ratio, $cv$)
- Age-related patterns of advanced microstructural measures (FR, ${d}_a$, MTSat, g-ratio)
- Puberty-related patterns of advanced microstructural measures
- Sex-by-puberty interactions of advanced microstructural measures
- Relationship between microstructural predictors of conduction velocity (FR, ${d}_a$, MTSat, g-ratio, $cv$) and working memory performance (processing speed and working memory capacity) in a subset of 11 participants

For each aim, a formal model selection approach was used to determine whether specific terms should be included as predictors. The voxel-wise microstructural measure was set as the dependent variable for each mixed model (reference region = genu, sex = female). Age and sex were included as predictors in all models. Region, microstructural measure, and subject ID were set as random effects, to account for within-subject variation of microstructural measures within each region and measure. For aim iv), sex-by-puberty interactions were studied across measures (reference measure = FR) to determine the discriminating power of group differences compared to a commonly used surrogate of “axon density.”

For each primary aim tested, the fit of the original model was compared with subsequent models (including main effects and interaction terms) and selected based on lowest Akaike information criterion. Standardized model term coefficients are reported with 95% confidence interval (CI) bounds, as β [95% CI]. Marginal effects of mixed models were determined using *ggpredict*. Evidence for an association is signified when CIs do not cross zero and *P* < 0.005, to represent an even balance between type I error and discovery of effects (Benjamin et al. 2018).

Apparent axon diameter model fit estimates for the full and truncated distributions are summarized in Supplementary Fig. S1a and b, using voxel-wise estimates of log likelihood. G-ratio estimates computed using AVF derived from either CHARMED or NODDI are summarized in Supplementary Fig. S1c. Estimates of ${d}_a$ from the full AxCaliber Poisson distribution and g-ratio predicted from NODDI were selected for further analysis due to lower regional variability in model fit and absolute values, respectively (see Supplementary Section 9.1 for further details).

## Results

### Developmental characteristics

Participant characteristics and their relationship with age are summarized in Table 1 and Supplementary Fig. S2. We observed a significant positive relationship between age and total, adrenal and gonadal pubertal stage, as well as BMI. There was no evidence for a relationship between age and total SDQ score. In terms of working memory, we observed a negative relationship between age and hit reaction time, whereby older adolescents had faster reaction times. In contrast, there was no significant relationship between age and ${d}^{\prime}$. There was no effect of age on RMS displacement for either the fixed or variable diffusion time protocols.

The correspondence between self and parent ratings for both pubertal development and difficulties scores was high and followed similar patterns with age (Supplementary Fig. S2). Parent-reported pubertal stage was correlated with self-report for overall pubertal stage (PDSS: *R*^2^ = 0.62, *P* < 0.001), adrenal pubertal stage (PDSA: *R*^2^ = 0.46, *P* < 0.001), and gonadal pubertal stage (PDSG: *R*^2^ = 0.70, *P* < 0.001). Parent-reported total SDQ score was correlated with self-report (*R*^2^ = 0.30, *P* < 0.001). Since the number and age of self-reporting participants differed from parent report (*N* = 37 vs. *N* = 50), and due to the high correspondence between self-report and parent-reported scores, subsequent analyses used parent-reported data for consistency across the full age distribution.

### Microstructural variation across the corpus callosum

**Fig. 2:**
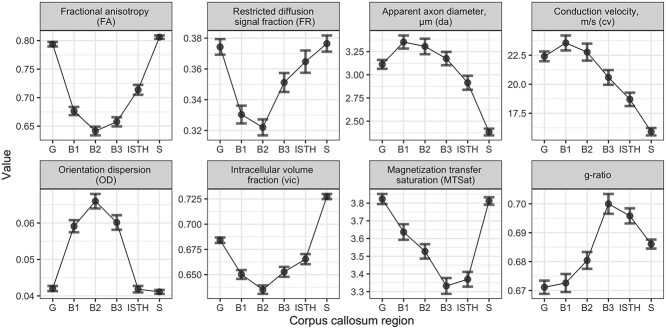
Regional profiles of microstructural measures across the corpus callosum. Error bars denote 95% confidence intervals. B1 = anterior body; B2 = midbody; B3 = posterior body; G = genu; ISTH = isthmus; S = splenium.

The regional microstructural profiles across the corpus callosum are summarized in Table 2 as descriptive statistics and visualized in Fig. 2. The results from the best-fitting model are presented in Supplementary Table S2.

**Table 2:** Summary statistics for microstructural measures across the corpus callosum.

Region	Med	MAD	Med	MAD	Med	MAD	Med	MAD
	FA		FR		${d}_a$ (μm)		$cv$ (m/s)	
G	0.80	0.06	0.37	0.09	3.17	0.69	22.7	5.0
B1	0.69	0.07	0.33	0.05	3.37	0.62	23.6	4.7
B2	0.65	0.08	0.32	0.06	3.23	0.75	22.0	4.6
B3	0.67	0.07	0.35	0.06	3.10	0.72	20.4	4.9
ISTH	0.74	0.08	0.36	0.10	2.87	0.75	19.0	4.8
S	0.81	0.06	0.38	0.12	2.35	0.62	15.6	4.3
	OD		${v}_{ic}$		MTSat		g-ratio	
G	0.04	0.005	0.68	0.05	3.86	0.46	0.67	0.04
B1	0.05	0.015	0.65	0.05	3.63	0.42	0.67	0.03
B2	0.06	0.017	0.63	0.05	3.54	0.39	0.68	0.04
B3	0.06	0.014	0.65	0.06	3.32	0.42	0.71	0.04
ISTH	0.04	0.004	0.66	0.06	3.38	0.49	0.69	0.04
S	0.04	0.005	0.73	0.06	3.80	0.43	0.69	0.04

Note: Values reported are median (med) and median absolute deviation (MAD). B1, anterior body; B2, midbody; B3, posterior body; $cv$, predicted conduction velocity, in m/s; ${d}_a$, apparent axon diameter, in μm; FR, restricted diffusion signal fraction; G, genu; ISTH, isthmus; MTSat, magnetization transfer saturation; OD, orientation dispersion; S, splenium; ${v}_{ic}$, intracellular volume fraction.

We observed that FA, FR, and ${v}_{ic}$ exhibited a high-low-high profile across the 6 regions, with a minimum in the mid body and maximum in the splenium. Similarly, MTSat exhibited a high-low-high profile, with a minimum in the posterior body, and peaking in the genu and splenium. In contrast, ${d}_a$ and $cv$ exhibited a low-high-low profile, where values were highest in the anterior body and lowest in the splenium. OD exhibited a low-high-low profile, peaking in the midbody. G-ratio followed an inverse pattern to MTSat, peaking in the posterior body, with higher values in posterior versus anterior regions.

### Advanced measures of axonal and myelin microstructure are associated with age and pubertal stage

**Fig. 3:**
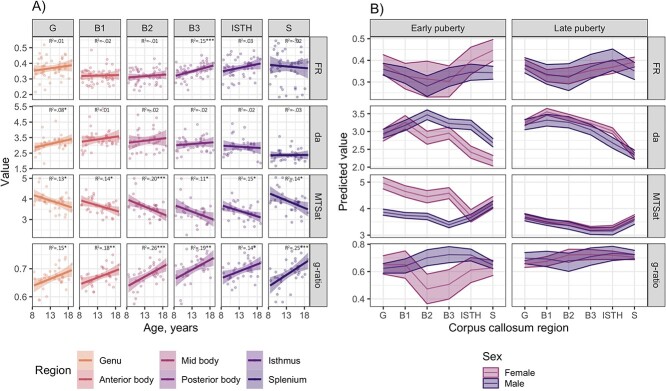
Microstructural correlates of age, sex, and pubertal stage. A) Relationship between advanced estimates of microstructure and age. Significant age relationships determined by a linear model are indicated by: ^*^p<.05; ^**^p<.01; ^***^p<.005. B) Marginal effects for sex-by-adrenal pubertal stage interactions, adjusted for age. ${d}_a$ = apparent axon diameter, in μm; FR = restricted diffusion signal fraction; MTSat = magnetization transfer saturation.

Age-related patterns of microstructural measures are presented in Fig. 3A. Age was positively associated with ${d}_a$ and g-ratio and negatively associated with MTSat. Models including microstructural measure, region, and age as interaction terms were preferred, suggesting that age relationships differed between the segments for some or all microstructural measures. We observed a significant effect of age on MTSat, β [95% CI] = −.14 [−0.23, −0.06], *P* = 0.002. We observed a significant age-by-region interaction for ${d}_a$ in the genu, β [95% CI] = 0.08 [0.04, 0.11], *P* < 0.001, and for g-ratio in the splenium, β [95% CI] = 0.11 [0.08, 0.15], *P* < 0.001.

When additionally modeling the effects of pubertal stage on microstructure (with age and sex as fixed effects), we observed a significant relationship between PDSA with MTSat, β [95% CI] = −0.37 [−0.56, −0.17], *P* < 0.001, and g-ratio, β [95% CI] = 0.03 [0.01, 0.05], *P* = 0.002.

### Sex differences in axon diameter and myelin content are most apparent in early puberty

The results for regional sex and puberty interactions are presented in Table 3 and Fig. 3B. The splenium of the corpus callosum showed the strongest cross-measure and cross-region relationship between sex and puberty. Models including adrenal pubertal stage (PDSA) as a predictor were preferred over gonadal (PDSG) and total (PDSS) pubertal stage. For ${d}_a$, we observed a significant effect of sex, and sex by PDSA interaction, described by males having a larger apparent axon diameter than females, most observable in the earlier stages of adrenarche in the midbody, isthmus, and splenium. For MTSat, we observed a significant effect of sex, and sex by PDSA interaction, described by females having higher myelin content than males, most observable in the earlier stages of adrenarche in anterior regions (genu and body). For g-ratio, we observed a significant effect of sex, described by males having higher g-ratio than females in mid regions. The reported findings were not influenced by subject motion (Supplementary Table S3). Overall, after adjusting for age, sex differences in early adrenarche were largest in posterior regions of the corpus callosum for axon-sensitive measures and in anterior regions of the corpus callosum for myelin-sensitive measures.

**Table 3:** Results for sex-by-pubertal stage interactions for advanced microstructural measures.

Effect		Region	β [95% CI]			*P*-value
Apparent axon diameter (${d}_a$), μm						
	Sex					
		B1	−0.38	−0.62	−0.13	**0.002**
		B2	0.19	−0.08	0.45	0.17
		B3	−0.20	−0.47	0.06	0.13
		ISTH	−0.12	−0.37	0.14	0.38
		S	0.70	0.52	0.89	**9.1E−14**
	Sex ^*^ puberty					
		B1	0.21	−0.04	0.46	0.10
		B2	−0.50	−0.77	−0.23	**2.9E−04**
		B3	−0.26	−0.53	0.01	0.06
		ISTH	−0.83	−1.08	−0.57	**1.4E−10**
		S	−0.64	−0.83	−0.46	**9.4E−12**
Magnetization transfer saturation (MTSat)						
	Sex					
		B1	−0.09	−0.36	0.18	0.50
		B2	0.01	−0.29	0.29	1.00
		B3	−0.44	−0.73	−0.15	**0.003**
		ISTH	0.12	−0.16	0.40	0.40
		S	0.69	0.49	0.89	**1.7E−11**
	Sex ^*^ puberty					
		B1	0.10	−0.18	0.37	0.50
		B2	−0.13	−0.42	0.16	0.39
		B3	0.23	−0.07	0.52	0.13
		ISTH	−0.91	−1.18	−0.64	**5.3E−11**
		S	−0.96	−1.16	−0.76	**<2e−16**
g-ratio						
	Sex					
		B1	−0.13	−0.40	0.14	0.34
		B2	0.24	−0.05	0.53	0.11
		B3	0.33	0.03	0.62	0.03
		ISTH	0.01	−0.27	0.29	0.94
		S	0.39	0.19	0.59	**1.5E−04**
	Sex ^*^ puberty					
		B1	0.36	0.09	0.64	0.01
		B2	−0.05	−0.35	0.24	0.72
		B3	−0.25	−0.55	0.04	0.09
		ISTH	0.06	−0.21	0.33	0.64
		S	−0.08	−0.29	0.12	0.41

Note: Results were computed using a linear mixed effect model (reference labels: measure = FR; region = genu; sex = female). Bold values indicate *P* < 0.005. B1, anterior body; B2, midbody; B3, posterior body; G, genu; ISTH, isthmus; S, splenium.

### Microstructural predictors of conduction velocity are associated with working memory

**Fig. 4:**
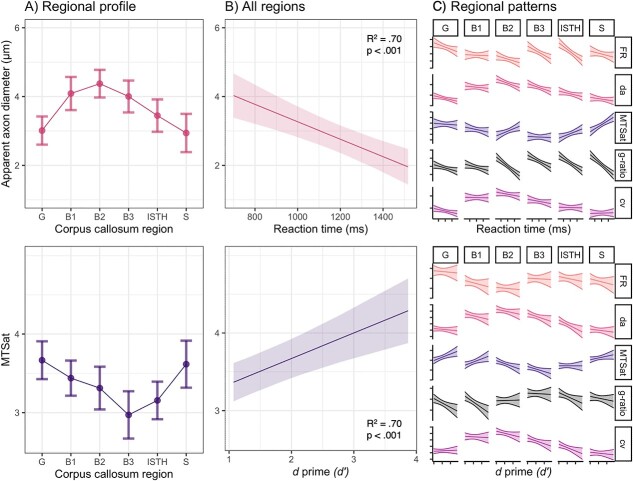
Relationship between microstructural predictors of conduction velocity and working memory. A) Regional profiles of apparent axon diameter (${d}_a$) and magnetization transfer saturation (MTSat) across the corpus callosum. Marginal effects for the relationship between working memory and microstructure across (B) all regions of the corpus callosum, and (C) individual regions of the corpus callosum. Predicted values estimated using marginal effects of linear mixed models were adjusted for age and sex. Raw unadjusted regional associations are visualized in Supplementary Figs S4 and S5.

The results for the relationship between advanced microstructural measures and working memory performance are presented in Table 4 and Fig. 4. Across all regions of the corpus callosum, ${d}_a$ was negatively associated with hit reaction time, after adjusting for age, consistent with the fact that larger axons conduct faster (Fig. 4B, top panel). Similar patterns were observed for $cv$. There was evidence for a positive relationship between MTSat and ${d}^{\prime}$ (Fig. 4B, bottom panel) and a negative relationship between g-ratio and ${d}^{\prime}$.

**Table 4:** Relationship between advanced microstructural measures and working memory performance.

Measure		Hit reaction time						*d* prime (${d}^{\prime}$)					
		*R* ^2^		β [95% CI]			*P*-value	*R* ^2^		β [95% CI]			*P*-value
FR		0.39		0.01	−0.17	0.20	0.88	0.38		−0.06	−0.17	0.06	0.32
${d}_a$		0.70		−0.63	−0.88	−0.38	**1.3E−04**	0.63		−0.02	−0.22	0.18	0.85
MTSat		0.65		−0.34	−0.69	0.02	0.09	0.70		0.57	0.36	0.78	**1.1E−04**
g-ratio		0.75		0.20	−0.25	0.66	0.40	0.75		−0.48	−0.74	−0.21	**0.005**
$cv$		0.65		−0.45	−0.68	−0.22	**0.001**	0.60		0.03	−0.17	0.23	0.80

Note: Summary statistics for each microstructural measure was estimated from linear mixed models, adjusted for age and sex. Conditional *R*^2^ summarizes variance explained by each model, including estimates from both fixed and random effects. Bold values denote *P* < 0.005.

Regional variation in the relationship between hit reaction time and microstructure was only apparent for MTSat and g-ratio (Fig. 4D), reflected as a positive relationship with MTSat in the splenium, β [95% CI] = 0.41 [0.26, 0.57], *P* < 0.001, and a negative relationship with g-ratio in the isthmus, β [95% CI] = −0.35 [−0.50, −0.20], *P* < 0.001, and splenium, β [95% CI] = −0.48 [−0.73, −0.24], *P* = 0.003. There were no significant interactions between region and ${d}^{\prime}$ for any of the advanced microstructural measures.

## Discussion

This study reports *in vivo* measurements of apparent axon diameter in healthy developing children and adolescents. Combined with a rich array of microstructural features, our findings reveal age- and puberty-related differences in axon- and myelin-sensitive measures.

Regional patterns of microstructure were in line with previously reported profiles across the corpus callosum in child and adolescent populations. Across the AP gradient, FA and ${v}_{ic}$ followed a known high-low-high profile, and OD followed a low-high-low profile (Bjornholm et al. 2017; Genc, Malpas, et al. 2018a; Garic et al. 2021). Similar to ${v}_{ic}$, FR followed a high-low-high pattern. Together, this suggests that axons are more densely packed in the genu and splenium compared to the midbody. The higher OD in the body of the corpus callosum likely explains its slightly lower FA (Pierpaoli et al. 1996; Nilsson et al. 2012). Apparent axon diameter followed a low-high-low pattern, peaking in the body and reaching a local minimum in the splenium, a region known to have a high proportion of small diameter axons (Aboitiz et al. 1992; Barazany et al. 2009; Alexander et al. 2010; Dyrby et al. 2013; Sepehrband et al. 2016)—although it is important to note (as will be discussed in Limitations and Future Directions section), despite the *trend* replicating the histological results from Aboitiz et al. (1992), the absolute values of axon diameter are overestimated. In terms of myelin content, MTSat followed a previously reported pattern (Bjornholm et al. 2017) described by a thinner myelin sheath in bigger axons (Paus and Toro 2009). Estimates of g-ratio (median g-ratio = 0.68) were in line with the optimal g-ratio observed *ex vivo* (Rushton 1951) and *in vivo* adult MRI studies (Ellerbrock and Mohammadi 2018; Thapaliya et al. 2018). Overall, the developmental profiles of axonal and myelin microstructure suggest a high density of large axons with thin myelin sheaths in the body of the corpus callosum and densely packed small diameter axons with relatively thicker myelin sheaths in the genu and splenium (Paus and Toro 2009).

Our findings of a positive relationship between age and apparent axon diameter in the genu of the corpus callosum would be consistent with a prolonged radial axonal growth contributing to previously reported developmental changes in white matter microstructure. Together with recent *in vivo* MRI observations in adults, this might suggest that the genu undergoes prolonged radial axonal growth in childhood followed by selective axonal loss in later adulthood (Fan et al. 2019).

Our *in vivo* findings of a sex-by-puberty interaction in posterior callosal axon diameter suggest that males have a larger apparent axon diameter than females in early adrenarche. Early work in rodent models have demonstrated age-related increases in axon diameter in the splenium (Kim and Juraska 1997), with larger axons in males than females in early puberty compared with prepuberty and late adolescence. We also found that g-ratio was higher in males compared to females, followed by a positive relationship with age. Experimental models have revealed lower axon diameter and lower g-ratio in the splenium of castrated male rats compared with non-castrated males (Pesaresi et al. 2015), implicating testosterone in modulating the radial growth of the axon. In typical neurodevelopment, MRI estimates of g-ratio rapidly decrease in early life (Dean 3rd et al. 2016) and appear to stabilize across selective white matter tracts in childhood and adolescence (Geeraert et al. 2019) and across the lifespan (Berman et al. 2017). As g-ratio increases as a function of axon diameter (Pesaresi et al. 2015; Mancini et al. 2021), our results in the context of previously reported *ex vivo* findings implicate rising testosterone levels in inducing structural reorganization of corpus callosum microstructure (Maninger et al. 2009; Schulz et al. 2009).

In contrast, we found that females had higher myelin content in anterior regions during early adrenarche, followed by a negative relationship with age. Although myelination is known to continue throughout childhood and adolescence (Yakovlev and Lecours 1967), developmental MRI studies using myelin-sensitive contrasts have produced heterogenous reports of constant (Geeraert et al. 2019), decreasing (Perrin et al. 2009; Pangelinan et al. 2016), and increasing (Vanes et al. 2020) estimates of myelin content with age. These inconsistencies could be due to the differential sensitivity of various models and acquisition techniques (Mancini et al. 2020). Our specific observations in the corpus callosum may be due to pubertal influences on axonal caliber, where increases in axonal caliber due to testosterone influx can reduce the number of axons per unit volume and lead to an apparent reduction in the myelination index (Perrin et al. 2008). Relatedly, the disproportionate rate of axonal and myelin development in male adolescence (Perrin et al. 2008) could lead to an increase of g-ratio, if the radial axonal growth outpaces myelin thickening (Paus and Toro 2009). Berman et al. (2017) showed no age dependence of g-ratio across the lifespan, using FA as a surrogate marker of axonal volume fraction. However, comparing patterns of g-ratio between studies can be difficult due to the various modeling and acquisition approaches used to determine axonal and myelin volume fraction, as DTI-based estimation of AVF can conflate multiple tissue properties and lead to inaccurate estimates (Campbell et al. 2018).

Longitudinal *in vivo* MRI studies incorporating hormonal sampling may provide clues as to why the observed sex differences in apparent axon diameter and myelin content are most observable in early adrenarche (Vijayakumar et al. 2018). Previous DTI studies have revealed that higher testosterone levels in males are linked to higher FA in the corpus callosum (Herting et al. 2012), whereas increases in testosterone levels are associated with female FA increases in the corpus callosum from early to mid-puberty (Ho et al. 2020). It is likely that sex differences in microstructure are present from the peri-natal activation of the HPA axis (Schulz et al. 2009) and that peri-pubertal surges of testosterone which accompany the reactivation of the HPA axis initiate a new period of structural remodeling. Our observations of a sex-by-pubertal stage interaction in *specific* microstructural properties underlying previous DTI findings suggest that increasing testosterone levels resolves sex differences in axonal diameter and myelin content when puberty is close to completion. Further work combining longitudinal hormonal sampling with advanced microstructural MRI is required to confirm these associations.

Finally, our secondary analysis of microstructural predictors of conduction velocity indicates a link between working memory and micrometer scale tissue properties in a developmental population, extending on previously found relationships using DTI measures (Fryer et al. 2008; Ferrer et al. 2013; Krogsrud et al. 2018). Previous *in vivo* MRI studies have linked higher FA with developmental improvements in working memory capacity and processing speed (Fryer et al. 2008; Ferrer et al. 2013). Our findings *specifically* shed light on the white matter properties that could underlie these associations. Our observation of faster processing speed coupled with larger apparent axon diameter and higher predicted conduction velocity is likely driven by larger diameter axons (e.g. in the midbody) that support rapid information transfer of sensorimotor stimuli (Gillespie and Stein 1983; Horowitz et al. 2015). In contrast, we observed that greater working memory capacity was closely linked with higher myelin content and lower g-ratio, which could be mediated by densely packed small diameter axons with higher myelin content (e.g. in the genu and splenium) that carry diverse signals that favor the quantity of information over speed (Alexander et al. 2010). Together, our findings suggest that specific microstructural properties, such as axon diameter and myelin content, facilitate gains in rapid and diverse interhemispheric neural transmission over child and adolescent brain development. Future work should confirm these preliminary findings in a larger sample size.

### Limitations

We present a novel and first-of-its-kind application of ultra-strong gradients. Despite the relatively small sample size (i.e. compared to other microstructural developmental studies), our results closely mirror *ex vivo* findings, suggesting that ultra-strong gradient MRI provides enhanced sensitivity to microstructural developmental patterns than more commonly available hardware (Chamberland et al. 2019; Chamberland et al. 2020; Genc et al. 2020; Raven et al. 2020). There are, however, limitations to our study that we wish to discuss.

*In vivo* MRI estimation of axon diameter from dMRI is challenging. First, estimates are constrained by the resolution limit (Nilsson et al. 2017), which may result in the oversampling of larger axon diameters and biased absolute estimates (Paquette et al. 2020). Second, it is known that OD can influence axon diameter estimates derived from the AxCaliber model (Drobnjak et al. 2016). As shown by others (Mollink et al. 2017), OD is non-negligible in the midline corpus callosum. We previously reported (in an independent population) that OD, although low overall, increased with age in the midline callosum (Genc, Malpas, et al. 2018a), and so indeed our apparent axon diameter estimates in regions of higher OD must be interpreted carefully. However, in the current study, the age-related patterns of OD are different from those of ${d}_a$, suggesting little influence on our main findings.

Third, acquisition considerations such as fixing the gradient pulse duration and omitting the apparent extra-axonal kurtosis can introduce biases into parameter estimation (Lee et al. 2018). Fourth, as shown by Palombo et al. (2017), the inner diameter of glial processes could be larger than that of neuronal processes and therefore, with increased myelin, there may be extra glial processes that could positively bias the estimates of axon diameter.

### Future opportunities

In the estimation of axon diameter, future work could consider employing alternative approaches, such as the powder-averaging approach of Veraart et al. (2020), which accounts for OD, although this so-called power-law approach requires much higher *b*-values than used here. We further note that for extension to other regions of white matter, this orientational-average approach may preclude the possibility of quantifying within-fasciculus properties in regions of crossing fibers.

One should consider the impact of other pruning-related processes occurring in puberty/mid-adolescence (Bartzokis et al. 2004; Bartzokis 2005), which, in addition to increases in myelination, include axonal and dendritic pruning. These latter processes may lead to an increase in the extra-axonal space. We note that the modeling approaches we use here assign diffusion in the intra-axonal space to be restricted, and extra-axonal space to be hindered, the latter being modeled with a time-dependent zeppelin (De Santis et al. 2016). Thus, if pruning creates a larger extra-axonal space with hindered diffusion, it should not impact the estimate of the intra-axonal signal fraction and inner diameter. However, if these processes in some way lead to the formation of spaces between axons that are non-communicating or disconnected, such that diffusion is restricted within these spaces and which have comparable dimensions to the axons themselves, then this could potentially bias estimates of the intra-axonal space.

Separate to the challenges of estimating axon diameter, while the pubertal staging method used here has a high correspondence with hormone levels (Shirtcliff et al. 2009), future work should consider saliva sampling of adrenal and steroid hormones in combination with a larger sampled longitudinal MRI study design to confirm the theory of testosterone-induced axonal remodeling and biological sex (Herting and Sowell 2017; Ho et al. 2020).

## Conclusion

In a developmental sample of children and adolescents aged 8–18 years, our *in vivo* findings uncover: (i) age-related patterns of apparent axon diameter, myelin content, and g-ratio; (ii) a larger apparent axon diameter coupled with lower myelin content in males during early puberty; and (iii) an association between microstructural predictors of conduction velocity and working memory processing speed. Together, these findings support previous results from *ex vivo* models and confirm the critical role of developmental factors to axon and myelin microstructure over the human child and adolescent period.

## Supplementary Material

Genc_axondev_2020_supplementary_bhac515

## Data Availability

The data that support the findings of this study are available on request from the corresponding author. The data are not publicly available due to privacy or ethical restrictions.
