# Supplementary material for: Novel insights into axon diameter and myelin content in late childhood and adolescence
Source: Cereb Cortex. 2023 Jan 4;33(10):6435–48. doi: 10.1093/cercor/bhac515 (PMC10183755; doi:10.1093/cercor/bhac515)
Supplement: Genc_axondev_2020_supplementary_bhac515 [file genc_axondev_2020_supplementary_bhac515.docx]

1. **Supplementary**
   1. **Supplementary information**

*Axon diameter model considerations*

Apparent axon diameter model fit estimates are summarised in Figure S1a,b. For dMRI acquisition (2) with variable diffusion time, regional analysis revealed a poorer model fit (lower log likelihood) in the anterior body, β [95% CI] = -.21 [-.31, -.11], posterior body, β [95% CI] = -.25 [-.42, -.09], and splenium, β [95% CI] = -.40 [-.61, -.20], compared with the genu. When comparing the two distributions (full and truncated), we observed an interaction between distribution type and region in the splenium, β [95% CI] = -.10 [-.15, -.05], explained by a slightly poorer model fit of the truncated distribution in this region. For dMRI acquisition (3) with variable diffusion time, regional analysis of model fit across the two distributions (full and truncated) revealed a slightly poorer fit (higher AIC, lower log likelihood) in the splenium region, β [95% CI] = -.43 [-.65, -.22], compared to the mid body. When comparing the two distributions (full and truncated), there was evidence for a slightly poorer model fit for the truncated distribution across all regions, β [95% CI] = -.04 [-.07, -.02]. Based on these findings, subsequent analyses were performed with $d_{a}$ estimated using the full distribution.

In studying developmental patterns of microstructure, we restricted our analyses to metrics hypothesised to vary with age, sex and pubertal stage (FR, $d_{a}$, MTSat, g-ratio; see aims in section 2.5). However, as it is possible that variations in orientation dispersion could affect the apparent axon diameter estimates reported here, we explored how OD varies with age, sex and puberty. Confirmatory analysis revealed distinct patterns of OD to apparent axon diameter (Figure S3).

- 1. **Supplementary tables**

**Table S1:** Completion rates for advanced microstructural imaging acquisition.

| **Acquisition** | **Completed** (N) | **Included**  (N) | **Total time**  (min, sec) |
| --- | --- | --- | --- |
| MPRAGE | 50 | 50 | 5m 35s |
| Multi-shell dMRI |  |  |  |
| Protocol (1) | 50 | 48 | 16m 14s |
| Protocol (2) | 38 | 36 | 29m 14s |
| Protocol (3) | 12 | 12 | 16m |
| Multi-parametric mapping | 45 | 34 | 10m 31s |

Note: Only participants which underwent either axon diameter mapping protocol (2 or 3) were included in the current study. Following visual inspection for motion artefact, 2 dMRI and 11 MPM datasets were excluded.

**Table S2:** Summary of mixed model results for the analysis of regional patterns.

| **Effect** | **Metric** | **Region** |  | **β [95% CI]** | | |  | **p-value** |
| --- | --- | --- | --- | --- | --- | --- | --- | --- |
|  | FR |  |  | -.32 | -.47 | -.16 |  | **1.9E-04** |
|  | $d_{a}$ |  |  | -.25 | -.45 | -.05 |  | .02 |
|  | $cv$ |  |  | -.01 | -.21 | .20 |  | .96 |
|  | OD |  |  | -.77 | -.93 | -.62 |  | **5.7E-14** |
|  | $v_{ic}$ |  |  | -.47 | -.59 | -.35 |  | **1.3E-10** |
|  | MTSat |  |  | -.16 | -.42 | .10 |  | .24 |
|  | g-ratio |  |  | -.73 | -1.01 | -.46 |  | **5.3E-06** |
| Region |  |  |  |  |  |  |  |  |
|  |  | B1 |  | -1.21 | -1.29 | -1.12 |  | **< 2e-16** |
|  |  | B2 |  | -1.57 | -1.67 | -1.47 |  | **< 2e-16** |
|  |  | B3 |  | -1.40 | -1.50 | -1.31 |  | **< 2e-16** |
|  |  | ISTH |  | -.81 | -.90 | -.73 |  | **< 2e-16** |
|  |  | S |  | .15 | .08 | .22 |  | **3.4E-05** |
| Sex |  |  |  | .02 | -.02 | .06 |  | .38 |
| Age |  |  |  | .01 | .01 | .01 |  | .06 |
| Metric * region | FR |  |  |  |  |  |  |  |
|  |  | B1 |  | .75 | .64 | .85 |  | **< 2e-16** |
|  |  | B2 |  | 1.02 | .90 | 1.13 |  | **< 2e-16** |
|  |  | B3 |  | 1.16 | 1.04 | 1.27 |  | **< 2e-16** |
|  |  | ISTH |  | .75 | .65 | .86 |  | **< 2e-16** |
|  |  | S |  | -.10 | -.17 | -.02 |  | .01 |
|  | $d_{a}$ |  |  |  |  |  |  |  |
|  |  | B1 |  | 1.52 | 1.40 | 1.63 |  | **< 2e-16** |
|  |  | B2 |  | 1.80 | 1.67 | 1.92 |  | **< 2e-16** |
|  |  | B3 |  | 1.46 | 1.34 | 1.58 |  | **< 2e-16** |
|  |  | ISTH |  | .56 | .45 | .68 |  | **< 2e-16** |
|  |  | S |  | -1.03 | -1.12 | -.95 |  | **< 2e-16** |
|  | $cv$ |  |  |  |  |  |  |  |
|  |  | B1 |  | 1.43 | 1.29 | 1.56 |  | **< 2e-16** |
|  |  | B2 |  | 1.66 | 1.51 | 1.81 |  | **< 2e-16** |
|  |  | B3 |  | 1.06 | .92 | 1.21 |  | **< 2e-16** |
|  |  | ISTH |  | .15 | .01 | .28 |  | .03 |
|  |  | S |  | -1.32 | -1.42 | -1.22 |  | **< 2e-16** |
|  | OD |  |  |  |  |  |  |  |
|  |  | B1 |  | 2.22 | 2.11 | 2.32 |  | **< 2e-16** |
|  |  | B2 |  | 2.98 | 2.86 | 3.09 |  | **< 2e-16** |
|  |  | B3 |  | 2.47 | 2.36 | 2.59 |  | **< 2e-16** |
|  |  | ISTH |  | .81 | .70 | .92 |  | **< 2e-16** |
|  |  | S |  | -.20 | -.28 | -.12 |  | **5.0E-07** |
|  | $v_{ic}$ |  |  |  |  |  |  |  |
|  |  | B1 |  | .66 | .55 | .76 |  | **< 2e-16** |
|  |  | B2 |  | .75 | .64 | .87 |  | **< 2e-16** |
|  |  | B3 |  | .91 | .79 | 1.02 |  | **< 2e-16** |
|  |  | ISTH |  | .54 | .43 | .65 |  | **< 2e-16** |
|  |  | S |  | .55 | .47 | .63 |  | **< 2e-16** |
|  | MTSat |  |  |  |  |  |  |  |
|  |  | B1 |  | .87 | .75 | .99 |  | **< 2e-16** |
|  |  | B2 |  | 1.06 | .93 | 1.19 |  | **< 2e-16** |
|  |  | B3 |  | .52 | .40 | .65 |  | **5.0E-16** |
|  |  | ISTH |  | -.03 | -.14 | .09 |  | .64 |
|  |  | S |  | -.18 | -.26 | -.09 |  | **6.5E-05** |
|  | g-ratio |  |  |  |  |  |  |  |
|  |  | B1 |  | 1.24 | 1.12 | 1.36 |  | **< 2e-16** |
|  |  | B2 |  | 1.72 | 1.59 | 1.85 |  | **< 2e-16** |
|  |  | B3 |  | 2.03 | 1.90 | 2.15 |  | **< 2e-16** |
|  |  | ISTH |  | 1.40 | 1.29 | 1.52 |  | **< 2e-16** |
|  |  | S |  | .22 | .14 | .31 |  | **4.0E-07** |

Note: Bold values indicate p<.005. Reference labels: metric = FA; region = genu; sex = female. Abbreviations: B1 = anterior body; B2 = mid body; B3 = posterior body; $cv$ = predicted conduction velocity; $d_{a}$ = apparent axon diameter; FR = restricted diffusion signal fraction; G = genu; ISTH = isthmus; MTSat = magnetization transfer saturation; OD = orientation dispersion; S = splenium; $v_{ic}$ = intra-cellular volume fraction.

**Table S3:** Summary of mixed model results for the analysis of sex and pubertal stage, with and without motion as a covariate.

| **Effect** |  | **No motion** | |  | **Motion** | |
| --- | --- | --- | --- | --- | --- | --- |
|  |  | **F-value** | **p-value** |  | **F-value** | **p-value** |
| Metric |  | .83 | .49 |  | 2.18 | .12 |
| Region |  | 4.35 | **.002** |  | 2.96 | .02 |
| Sex |  | .01 | .99 |  | .01 | .99 |
| Puberty |  | .70 | .41 |  | .36 | .55 |
| Age |  | .72 | .40 |  | 2.21 | .15 |
| Motion |  | - | - |  | .13 | .72 |
| Metric * region |  | 150.40 | **< 2e-16** |  | 138.01 | **< 2e-16** |
| Metric * sex |  | 3.83 | .02 |  | 3.02 | .05 |
| Region * sex |  | 1.11 | .37 |  | 1.85 | .12 |
| Metric * puberty |  | 8.79 | **2e-04** |  | 3.66 | .03 |
| Region * puberty |  | 1.15 | .34 |  | .91 | .48 |
| Sex * puberty |  | .82 | .38 |  | .61 | .44 |
| Metric * region * sex |  | 11.98 | **< 2e-16** |  | 17.41 | **< 2e-16** |
| Metric * region * puberty |  | 11.96 | **< 2e-16** |  | 13.49 | **< 2e-16** |
| Metric * sex * puberty |  | 3.64 | .02 |  | 2.98 | .05 |
| Region * sex * puberty |  | .63 | .68 |  | .62 | .69 |
| Metric * region * sex * puberty |  | 16.55 | **< 2e-16** |  | 14.24 | **< 2e-16** |

Note: Motion parameters estimated using root mean-squared displacement (RMS) from *eddy*. Bold values indicate p<.005.

- 1. **Supplementary figures**


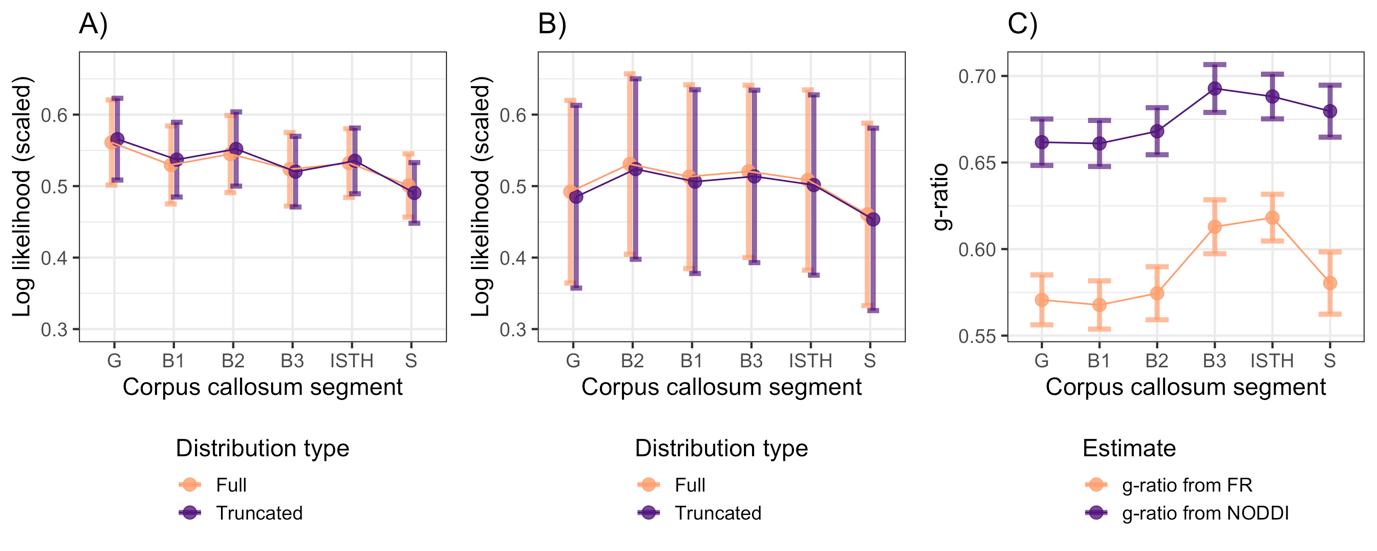


**Figure S1:** Parameter estimation across regions of the corpus callosum. AxCaliber model fit grouped by axon diameter distribution type for A) dMRI acquisition (2) with variable diffusion time, and B) dMRI acquisition (3) with variable diffusion time. C) G-ratio estimation grouped by axonal volume fraction estimation method from CHARMED (using FR) or from NODDI (using $v_{ic}$ and $v_{iso}$). Error bars denote 95% confidence intervals.

**
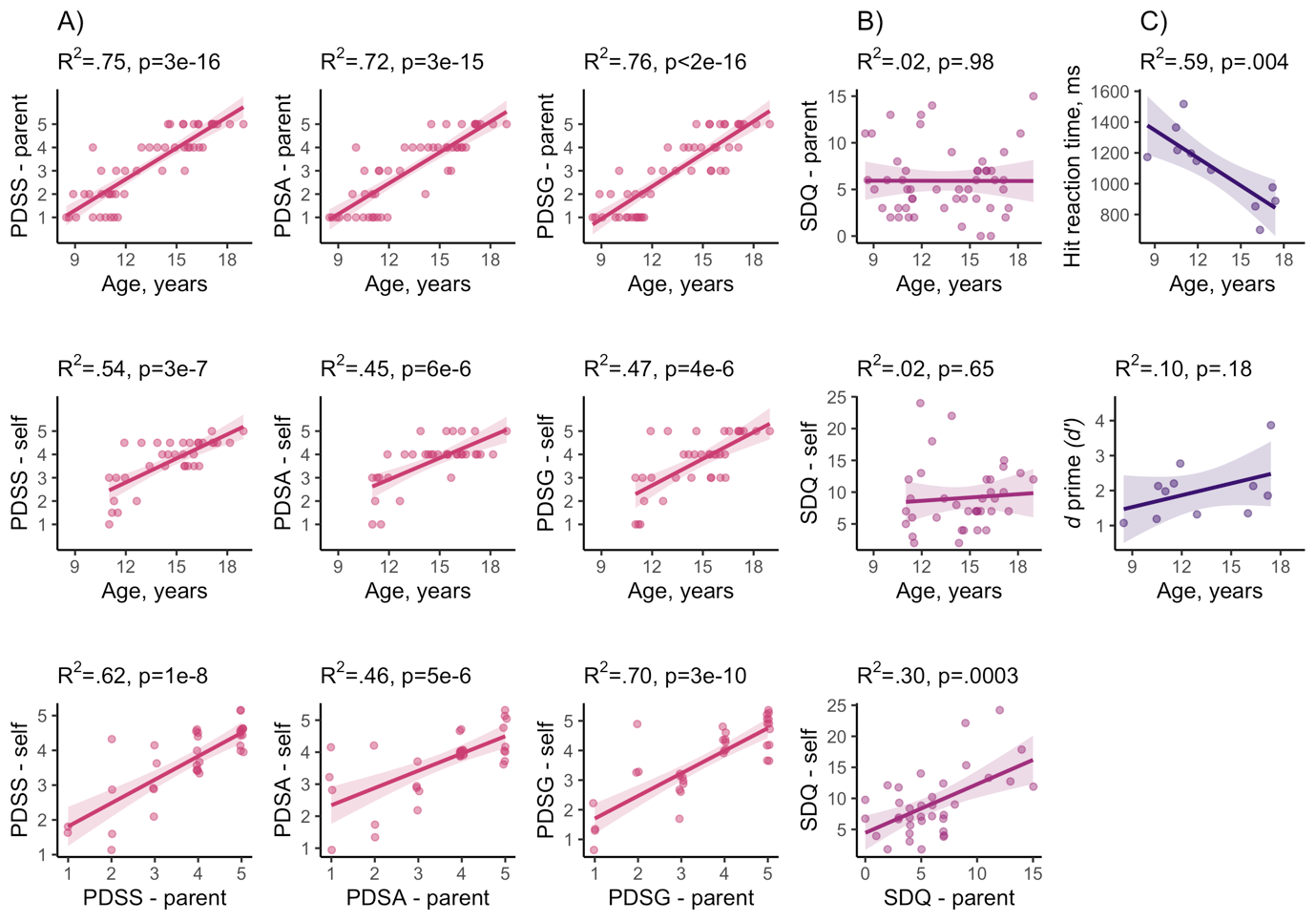
**

**Figure S2:** Developmental characteristics for A) pubertal stage, B) total SDQ score, and C) working memory performance. Age-related patterns of both parent and self-reported scores (top two rows), as well as concordance between parent and self-reported scores (bottom row), are visualised. Abbreviations: PDS = pubertal development scale; PDSA = adrenal PDS; PDSG = gonadal PDS; PDSS = total PDS; SDQ = strengths and difficulties questionnaire.


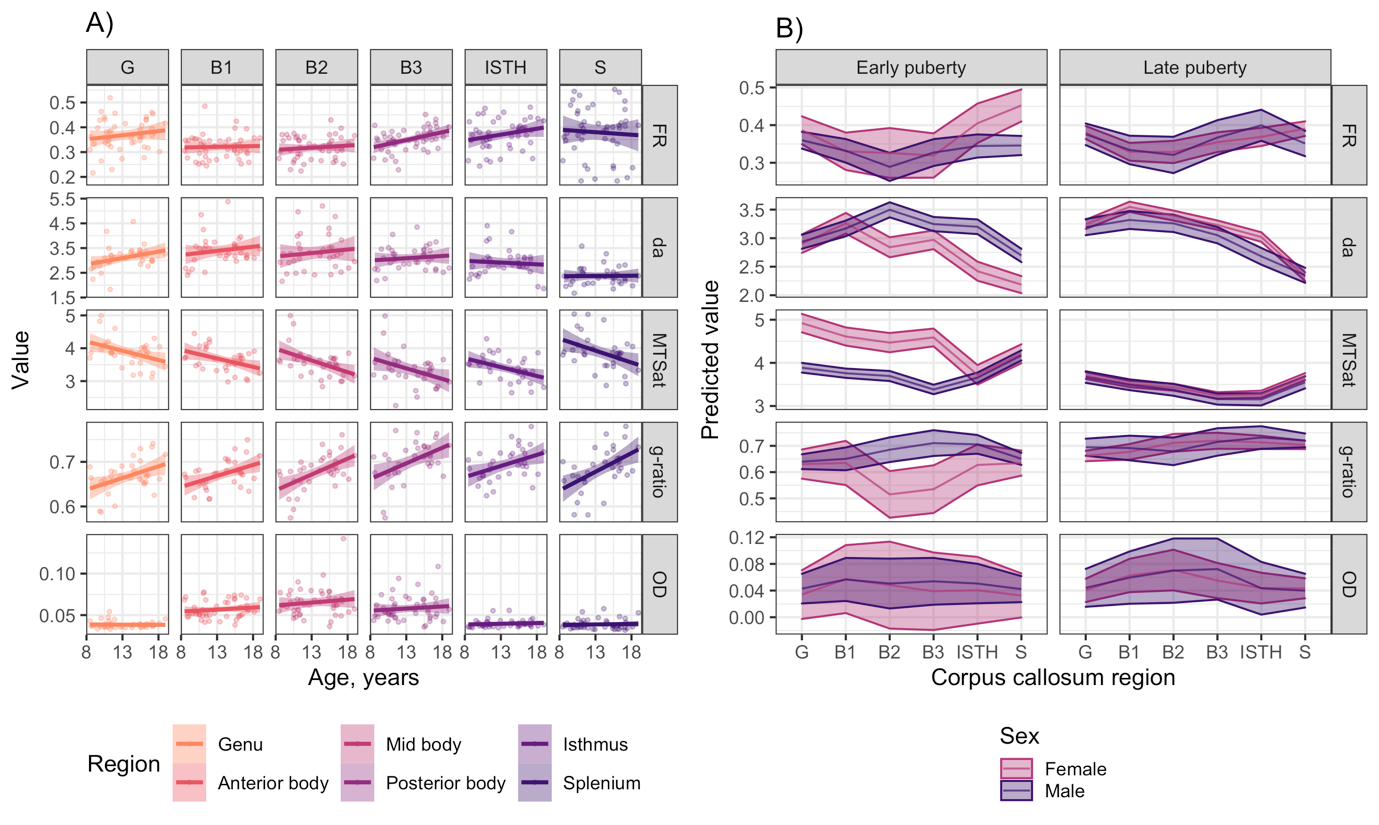


**Figure S3:** Microstructural correlates of age, sex and pubertal stage, including an estimate of orientation dispersion. A) Relationship between advanced estimates of microstructure and age. B) Marginal effects for sex by adrenal pubertal stage interactions, adjusted for age. Abbreviations: $d_{a}$ = apparent axon diameter, in µm; FR = restricted diffusion signal fraction; MTSat = magnetization transfer saturation; OD = orientation dispersion.


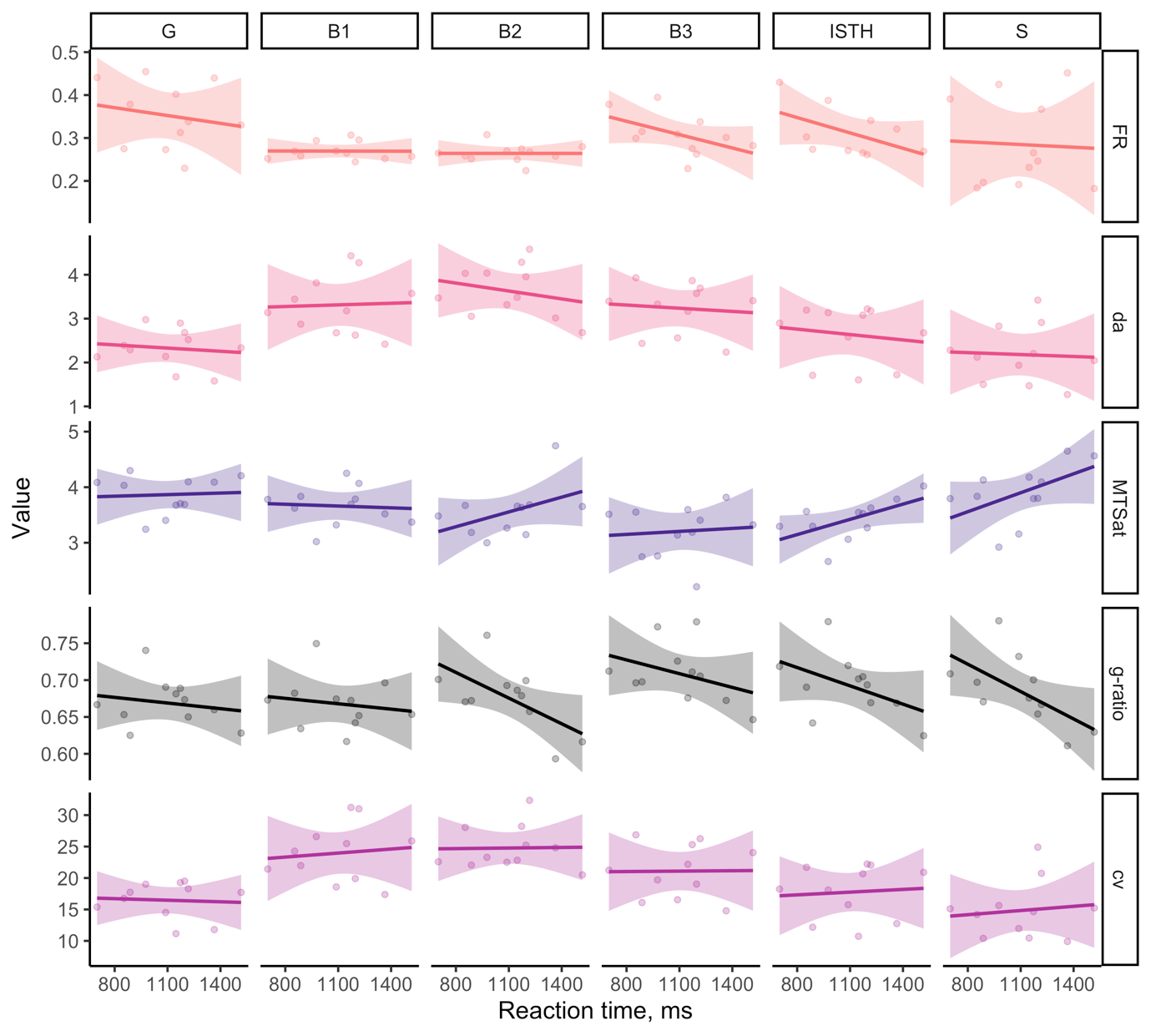


**Figure S4:** Raw associations between microstructural predictors of conduction velocity and cognitive processing speed. Abbreviations: B1 = anterior body; B2 = mid body; B3 = posterior body; $d_{a}$ = apparent axon diameter, in µm; FR = restricted diffusion signal fraction; G = genu; ISTH = isthmus; MTSat = magnetization transfer saturation; S = splenium.


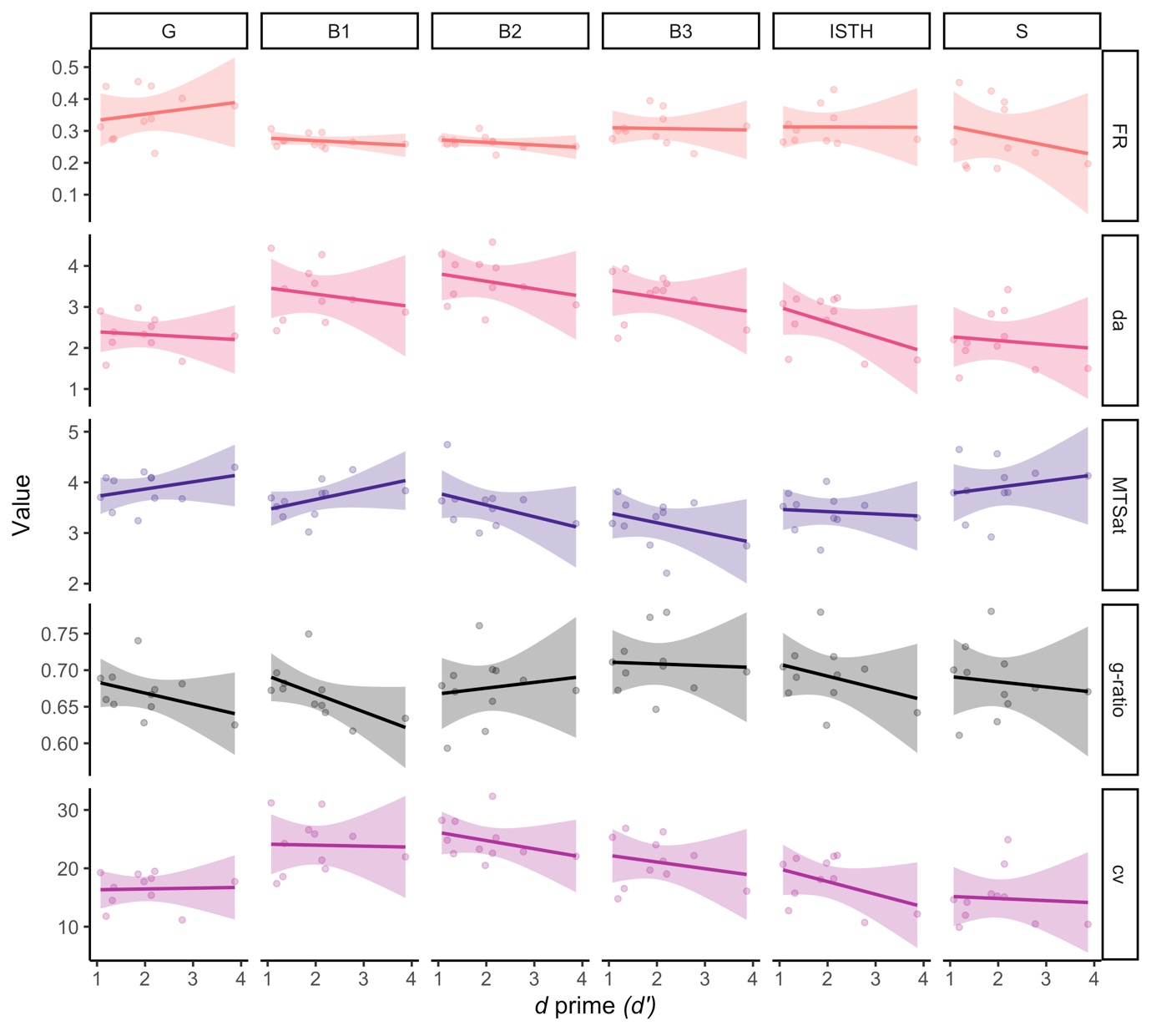


**Figure S5:** Raw associations between microstructural predictors of conduction velocity and working memory capacity. Abbreviations: B1 = anterior body; B2 = mid body; B3 = posterior body; $d_{a}$ = apparent axon diameter, in µm; FR = restricted diffusion signal fraction; G = genu; ISTH = isthmus; MTSat = magnetization transfer saturation; S = splenium.
